# Myocardial Ischemia–Reperfusion Injury—Mechanistic Insights and Novel Therapeutics

**DOI:** 10.3390/ijms27052106

**Published:** 2026-02-24

**Authors:** Dong-Yeon Han, Hyo-Suk Ahn, Hun-Jun Park

**Affiliations:** 1 Department of Medical Sciences, College of Medicine, The Catholic University of Korea, Seoul 06591, Republic of Korea; dyeon99@catholic.ac.kr; 2 Division of Cardiology, Uijeongbu St. Mary’s Hospital, College of Medicine, The Catholic University of Korea, Uijeongbu 11765, Republic of Korea; alaco0502@gmail.com

**Keywords:** myocardial infarction, ischemia–reperfusion injury, cardioprotection, vascular leakage, immune modulation, antioxidant therapy

## Abstract

Myocardial ischemia–reperfusion (I/R) injury remains a major contributor to infarct expansion and adverse cardiac remodeling despite advances in timely reperfusion therapy. Although restoration of blood flow is essential for myocardial salvage, the abrupt transition from ischemia to reperfusion paradoxically exacerbates cardiomyocyte injury through profound metabolic, ionic, and mitochondrial disturbances. Reperfusion should be viewed not simply as restoration of blood flow, but as a critical biological transition that converts ischemic stress into a self-amplifying injury network. Reperfusion induces excessive reactive oxygen species generation, calcium overload, endothelial barrier disruption, and dysregulated innate immune activation, which converge on mitochondrial dysfunction and diverse forms of cell death, including apoptosis, necroptosis, pyroptosis, and ferroptosis. Emerging evidence highlights that these pathological processes are tightly interconnected through damage-associated molecular pattern signaling, microvascular leakage, and inflammatory amplification, underscoring the limitations of single-target therapeutic approaches. This review summarizes the molecular and cellular mechanisms underlying myocardial I/R injury with a particular focus on oxidative stress, immune modulation, vascular integrity, and ferroptosis. We further discuss current and emerging cardioprotective strategies, including antioxidant therapies, modulation of neutrophil recruitment, microvascular leakage blockade, and anti-ferroptotic interventions. Finally, we address key translational challenges and future perspectives for developing integrated cardioprotective therapies aimed at improving clinical outcomes in acute myocardial infarction.

## 1. Introduction

Ischemic heart disease (IHD), encompassing conditions such as acute myocardial infarction (MI), continues to impose the greatest global burden in terms of mortality and long-term functional impairment [1]. The effects of IHD are usually attributable to myocardial ischemia or infarction, which results from restricted blood flow caused by coronary artery obstruction. In the setting of acute myocardial ischemia, timely re-establishment of myocardial perfusion represents the most effective intervention, typically pursued via thrombolytic therapy or primary percutaneous coronary intervention (PCI) [2]. However, in some cases, myocardial reperfusion itself can paradoxically contribute to cardiomyocyte (CM) injury, referred to as ischemia–reperfusion (I/R) injury [3,4]. The extent to which reperfusion induces additional cardiomyocyte death beyond ischemic injury remains controversial; reperfusion can nonetheless exacerbate myocardial damage through abrupt metabolic, inflammatory, and microvascular disturbances, highlighting the complex pathophysiology of reperfusion-associated injury [5]. Clinically, reperfusion-associated injury remains highly relevant in acute ST-segment elevation MI (STEMI), where restoration of coronary blood flow paradoxically initiates biological processes that can further propagate myocardial damage [6]. Despite decades of research, translating mechanistic insights from myocardial I/R injury into effective clinical therapies remains a major challenge. Although substantial progress has been made in understanding I/R injury mechanisms and exploring therapeutic approaches targeting these pathways, no effective intervention currently exists to prevent myocardial I/R injury. While numerous reviews have comprehensively described the mechanisms of myocardial I/R injury, many have focused on individual pathways, which may partly explain the persistent clinical failure of single-target therapies. Accordingly, this review focuses on the interconnected pathophysiological networks underlying myocardial I/R injury, emphasizing how metabolic disruption, inflammatory amplification, microvascular dysfunction, and regulated cell death collectively drive myocardial damage. To provide a perspective beyond existing literature, we highlight the integrated nature of I/R pathology, particularly the interplay among damage-associated molecular pattern (DAMP) signaling, microvascular leakage, and inflammatory amplification in promoting cardiomyocyte death. Furthermore, to address the limitations of conventional approaches, we propose a spatiotemporal three-step defense line framework that categorizes emerging cardioprotective strategies into: (1) a first defense line targeting microvascular leakage, (2) a second defense line focusing on the spatiotemporal modulation of inflammatory immune responses, and (3) a third defense line utilizing advanced antioxidant strategies to prevent non-apoptotic programmed cell death, including ferroptosis. Taken together, these in-sights help explain why previous single-target cardioprotective strategies have failed and provide a rationale for emerging multi-target therapeutic approaches, offering a roadmap for mitigating I/R-induced myocardial damage.

## 2. Pathophysiology of Myocardial I/R Injury

### 2.1. Biochemical and Metabolic Changes Within the Myocardium After I/R Injury

An acute blockage of the infarct-related coronary artery induces myocardial ischemia, establishing an area at risk (AAR) that can evolve into infarction if the occlusion is sustained. Interruption of oxygen and substrate delivery rapidly triggers profound biochemical and metabolic disturbances in the ischemic myocardium. During ischemia, reduced O_2_ shifts cellular metabolism from glucose oxidation to anaerobic glycolysis, and this ‘uncoupling’ results in the accumulation of cytosolic lactate leading to acidification with intracellular pH < 7.0 [7]. Intracellular acidification stimulates the Na^+^/H^+^ exchanger, resulting in an accumulation of cytosolic Na^+^ [8]. However, the absence of oxygen halts oxidative phosphorylation and impaired electron transport chain function, leading to decreased mitochondrial adenosine triphosphate (ATP) production. Ischemia-associated ATP depletion compromises Na^+^/K^+^-ATPase-mediated ion regulation, leading to Na^+^ and Ca^2+^ overload via dysregulated Na^+^/Ca^2+^ exchange. The ensuing ionic imbalance promotes water entry into the cytoplasm, causing cell swelling, enzymatic dysfunction, and nuclear chromatin condensation [9].

Although oxygen is essential for cellular survival, uncontrolled activity of oxygen-derived intermediates promotes pathological ROS accumulation, resulting in oxidative injury to organelles and key macromolecular constituents, including lipids, proteins, carbohydrates, and nucleic acids [10]. During the initial minutes of myocardial reperfusion, reintroduction of oxygenated blood to previously ischemic tissue precipitates a rapid surge in reactive oxygen species (ROS), including superoxide, hydrogen peroxide, hydroxyl radicals, and peroxynitrite. These highly reactive molecules disrupt cellular redox homeostasis, giving rise to oxidative stress through multiple enzymatic and subcellular sources, such as activation of xanthine oxidase and NADPH oxidase, electron leakage from the mitochondrial respiratory chain, and uncoupling of nitric oxide synthase (NOS) [11]. This oxidative stress induces the destruction of cell and organelle membranes by lipid peroxidation, and triggers DNA strand breakage, leading to activation of the nuclear enzyme poly(ADP-ribose) synthetase and depletion of the intracellular NAD+, and subsequent impairment of glycolysis, electron transport, and ATP production [12].

Iron is a redox-active metal that contributes to lipid peroxidation via two major mechanisms. First, iron released from the labile iron pool (LIP) promotes ROS accumulation through Fenton chemistry [13]. Second, iron serves as an essential cofactor for en-zymes such as lipoxygenases and NADPH oxidases, which directly participate in lipid peroxidation processes. It is reported that post-ischemic reperfusion can provoke the dysregula-tion of iron regulatory proteins (IRPs) and down-regulation of ferroportin-1 (FPN1), leading to intracellular iron accumulation and thereby worsening iron-dependent non-apoptotic programmed cell death [14]. Ferroptosis occurs through the lethal accumulation of lipid-based ROS by the Fenton reaction when glutathione (GSH)-dependent lipid peroxide repair systems are compromised [15].

### 2.2. The Role of Mitochondrial Permeability Transition Pore in I/R Injury

The mitochondrial permeability transition pore (mPTP) is a nonselective channel of the inner mitochondrial membrane, which has become a critical determinant of lethal reperfusion injury. Under resting conditions, mitochondrial matrix Ca^2+^ levels are tightly maintained at low concentrations; however, Ca^2+^ overload triggers rapid and transient Ca^2+^ uptake into mitochondria [16]. Mitochondrial Ca^2+^ is a key regulator of cellular energy metabolism, enhancing ATP production by activating pyruvate dehydrogenase and the major rate-limiting dehydrogenases of the tricarboxylic acid cycle, including isocitrate dehydrogenase and α-ketoglutarate dehydrogenase, while concurrently supporting oxidative phosphorylation. These actions are mediated by tightly regulated physiological Ca^2+^ signaling, allowing mitochondrial ATP output to dynamically adjust to cellular energy requirements. In contrast, excessive accumulation of Ca^2+^ within the mitochondrial matrix disrupts respiratory efficiency by increasing oxygen consumption, promoting electron leakage from the respiratory chain, and amplifying ROS generation, thereby predisposing mitochondria to permeability transition pore opening [17]. Activation of the mPTP compromises the proton-impermeable function of the inner mitochondrial membrane, leading to dissipation of the mitochondrial membrane potential and a transient increase in mitochondrial ROS production [18]. During myocardial ischemia, mPTP opening is restrained by the acidic intracellular environment. Upon reperfusion, rapid pH normalization together with mitochondrial Ca^2+^ overload and oxidative stress renders mPTP permissive to activation during the early reperfusion phase. The magnitude of mPTP opening governs cellular outcome, ranging from reversible injury to engagement of programmed cell death pathways or irreversible mitochondrial dysfunction. Therefore, the mPTP represents an important new target for cardioprotection during reperfusion [19].

### 2.3. Inflammatory Immune Responses in I/R Injury

I/R injury induces a complex inflammatory immune response in the absence of pathogenic stimuli, a process commonly referred to as sterile inflammation [20]. During ischemia, damage-associated molecular patterns (DAMPs), including peroxiredoxin, high mobility group box-1, nucleotides, purines, and nucleic acid fragments, are released and act as early triggers of inflammation, with their effects extending into the reperfusion phase [21,22]. Among these DAMP molecules, the S100A8/A9 heterodimer (calprotectin) serves as a major endogenous amplifier of sterile inflammation during myocardial I/R injury. Released predominantly from activated neutrophils and inflammatory monocytes, S100A8/A9 engages Toll-like receptor (TLR) 4 and receptor for advanced glycation end-product (RAGE) signaling, thereby reinforcing NF-κB-dependent transcription, endothelial adhesion molecule expression, and additional leukocyte recruitment. Through this feed-forward mechanism, calprotectin links early DAMP release to sustained innate immune activation and microvascular dysfunction during reperfusion [23]. During reperfusion, DAMPs—including calprotectin—bind innate immune receptors such as TLRs on leukocytes, leading to activation of inflammatory transcription factors including NF-κB and mitogen-activated protein kinases. These signaling cascades drive the production of cytokines, chemokines, adhesion molecules, matrix metalloproteinases (MMPs), iNOS, and NOX, exacerbating ischemic injury [24]. Neutrophils are rapidly recruited, short-lived immune cells that serve as early responders after MI and contribute to both inflammatory and reparative phases of cardiac injury. Following MI, signals such as DAMPs released by damaged cardiomyocytes guide neutrophil infiltration into the infarct zone, where oxidative activation pathways trigger the production of cytokines, proteases, and neutrophil extracellular traps (NETs). Proteases such as neutrophil elastase (NE) and MMPs directly incite CM death and cardiac tissue damage [25]. NETs and cytokines such as tumor necrosis factor-α (TNF-α) amplify the inflammatory cascade through inflammatory monocyte recruitment and pro-inflammatory macrophage signaling. Macrophages in turn exacerbate cardiac damage by secreting an array of pro-inflammatory cytokines including TNF-α and interleukins (IL)-6 and -12. These injury-associated pathways delineate a secondary therapeutic window during the early post-reperfusion phase, typically spanning the first 1–3 days after MI, when neutrophil-driven inflammatory amplification predominates before reparative immune programs become established [26]. Preclinical studies suggest that interventions targeting neutrophil recruitment or activation are most effective when applied within this early interval, whereas delayed or prolonged suppression may interfere with inflammation resolution and tissue repair. In parallel with local immune cell infiltration, systemic inflammatory responses are reflected by circulating leukocyte-derived biomarkers frequently assessed in ACS-STEMI patients, including elevated neutrophil-to-lymphocyte ratio (NLR), high-sensitivity C-reactive protein (hs-CRP), interleukin-6 (IL-6), and myeloperoxidase (MPO), which have been associated with adverse cardiac remodeling and poorer clinical outcomes [27].

Beyond leukocyte-mediated inflammation in both tissue and circulation, platelet-driven thrombo-inflammatory responses play a central role in myocardial I/R injury by promoting thrombo-inflammatory signaling, microvascular obstruction, and recurrent thrombo-ischemic events [28]. Activated platelets interact with neutrophils and monocytes through platelet–leukocyte aggregates, thereby amplifying inflammatory activation and contributing to adverse cardiac remodeling. Clinically, dual antiplatelet therapy (DAPT), including aspirin and P2Y12 receptor inhibitors, remains a cornerstone of post-AMI management and is essential for reducing recurrent thrombotic events, highlighting the translational importance of platelet-mediated pathways [29]. However, residual microvascular and inflammatory injury may persist despite optimal platelet inhibition [5]. Together, these findings emphasize that thrombotic and inflammatory pathways are tightly intertwined during myocardial I/R injury, providing a strong rationale for integrated therapeutic strategies.

### 2.4. Microvascular Dysfunction and Capillary Leakage in I/R Injury

Intercellular junctions between adjacent endothelial cells are composed of multiple macromolecular complexes that tightly regulate the passage of fluids, solutes, and cells under physiological hemostatic conditions [30]. However, the disruption of endothelial integrity leads to the extravasation of fluid and macromolecules as well as the extravasation of leukocytes with tissue inflammation caused by the release of inflammatory cytokines. The central structural and functional component of the adherens junction is vascular endothelial (VE)-cadherin, which is uniquely expressed in endothelial cells [31]. VE-cadherin mediates homophilic interactions between adjacent endothelial cells and assembles into multicomponent complexes with cytoplasmic binding partners, among which p120 catenin plays a critical role in stabilizing VE-cadherin at the plasma membrane [32]. The integrity of intercellular VE-cadherin complexes is tightly regulated by the activation status of the endothelial actomyosin cytoskeleton. Under homeostatic conditions, actomyosin bundles organize into robust cortical structures that reinforce adherens junction stability. In contrast, permeability-inducing stimuli drive actin reorganization and radial stress fiber formation, leading to redistribution of VE-cadherin complexes toward focal junctions and subsequent junctional destabilization, increasing vascular permeability.

Cardiac microvascular endothelial cells (CMECs) exhibit greater susceptibility to reperfusion injury than CMs and play a pivotal role in the early pathological events of I/R injury. ROS, including superoxide and its downstream metabolites, modulate vascular homeostasis and endothelial function through diverse signaling pathways. Notably, ROS generation reaches its maximum within the first 2–10 min following myocardial reperfusion. Excessive ROS disrupt endothelial barrier integrity by destabilizing junctional proteins that normally preserve tight intercellular contacts between CMECs, thereby promoting microvascular hyperpermeability [33]. Concomitantly, adhesion molecules including intercellular adhesion molecule 1 (ICAM-1), vascular cell adhesion molecule 1 (VCAM-1), and E-selectin are upregulated on CMECs, facilitating neutrophil adhesion, capillary plugging, and infiltration into the infarcted myocardium. Recruited neutrophils generate markedly higher levels of ROS than CMECs, thereby amplifying inflammatory signaling and exacerbating myocardial injury. Within the cardiac microenvironment, CMECs and CMs are closely juxtaposed and engage in dynamic crosstalk through paracrine signaling as well as direct cell–cell interactions under both physiological and pathological conditions [34]. During the early phase of reperfusion, injured CMECs release soluble proapoptotic mediators that promote apoptosis in neighboring CMs. Conversely, activated CMECs enhance endothelial nitric oxide synthase (eNOS) expression, which supports CM survival and preserves contractile function.

**Figure 1:**
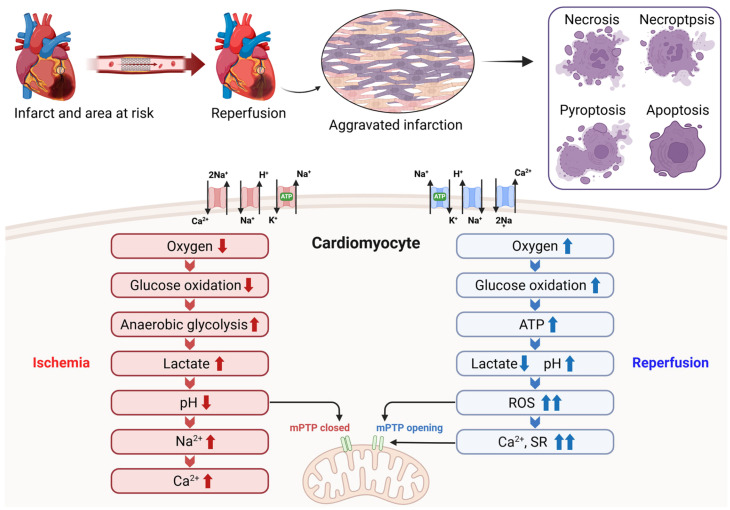
Metabolic and ionic disturbances underlying cardiomyocyte injury during myocardial I/R. During ischemia, reduced oxygen availability suppresses glucose oxidation and promotes anaerobic glycolysis, resulting in lactate accumulation, intracellular acidosis, and sodium and calcium overload while maintaining mPTP closure. Upon reperfusion, rapid oxygen reintroduction restores ATP production but paradoxically induces a burst of reactive oxygen species, calcium overload from the sarcoplasmic reticulum, and abrupt pH normalization, triggering mPTP opening. These events converge to activate multiple cardiomyocyte death pathways, including necrosis, necroptosis, pyroptosis, and apoptosis, thereby aggravating MI. Created with BioRender.com.

Ischemia and subsequent reperfusion impose distinct but interrelated metabolic, electrolytic, and mitochondrial stresses on cardiomyocytes, which converge to drive mitochondrial permeability transition and multiple forms of cell death. Rather than acting independently, these mechanisms converge into an interconnected injury network that amplifies myocardial damage during reperfusion. These integrated pathological processes are schematically summarized in Figure 1.

## 3. Emerging Therapeutic Strategies for Reducing Myocardial Ischemia–Reperfusion Injury

**Figure 2:**
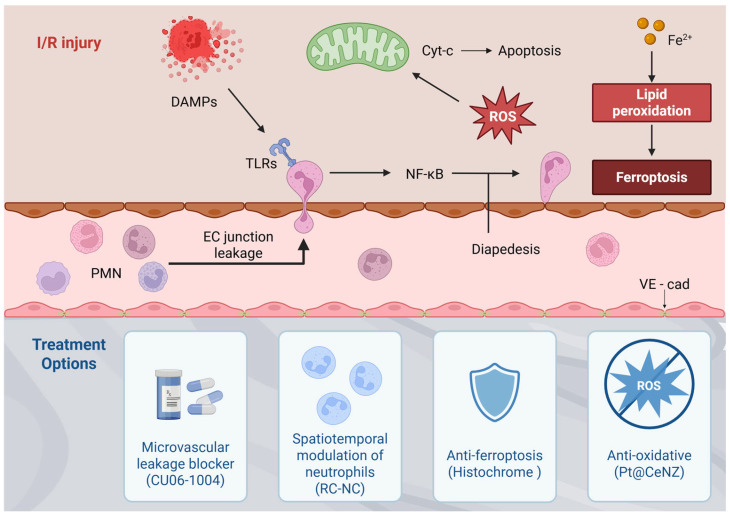
Integrated therapeutic strategies targeting myocardial I/R injury. DAMPs released after reperfusion activate Toll-like receptors and NF-κB signaling, promoting endothelial junction disruption, neutrophil diapedesis, oxidative stress, and lipid peroxidation-driven ferroptosis. Emerging cardioprotective approaches aim to mitigate these processes through microvascular leakage blockade, spatiotemporal modulation of neutrophil recruitment, antioxidant therapies, and anti-ferroptotic agents, thereby preserving myocardial and microvascular integrity. Created with BioRender.com.

Building on this integrated view of reperfusion injury, recent therapeutic strategies increasingly aim to modulate multiple interconnected pathological processes. In patients presenting with STEMI, rapid restoration of coronary blood flow remains the most established approach for mitigating acute ischemic injury and constraining MI size, most commonly achieved through thrombolytic therapy or primary PCI. Infarct magnitude is strongly influenced by the duration of ischemia, making reduction in the interval between symptom onset and primary PCI a central therapeutic objective. Despite these advances, no pharmacological strategy has yet proven effective in preventing lethal myocardial reperfusion injury in STEMI patients undergoing primary PCI. Moreover, interventions targeting individual pathogenic components of reperfusion injury—including oxidative stress, Ca^2+^ overload, abrupt pH normalization, and inflammation—have thus far yielded largely disappointing clinical outcomes [35]. The limited success of single-target strategies likely reflects several inherent challenges of myocardial I/R injury. First, multiple injury pathways operate simultaneously and compensate for one another, reducing the impact of targeting any single mechanism. Second, the rapid temporal evolution of reperfusion injury creates a narrow therapeutic window that is difficult to capture in clinical practice. Finally, substantial heterogeneity among STEMI patients—including ischemic duration, comorbidities, and microvascular status—further complicates translation of preclinical findings into consistent clinical benefit [36]. Accordingly, several emerging therapeutic strategies aimed at preventing lethal myocardial reperfusion injury have demonstrated promising results in small proof-of-concept studies. A comparative overview of currently used clinical therapies and emerging experimental strategies targeting myocardial I/R injury, together with their mechanistic focus and translational considerations, is summarized in Table 1. While Table 1 provides a comparative overview of clinical and emerging therapeutic strategies, Figure 2 summarizes the interconnected pathological networks that underpin these therapeutic targets.

### 3.1. First Defense Target—Microvascular Leakage Blockers

It has been convincingly demonstrated that myocardial edema and leukocyte infiltration constitute key determinants of reperfusion injury severity. On this basis, pharmacological strategies aimed at preserving microvascular endothelial barrier integrity represent a rational approach for the prevention and treatment of I/R injury. FX06 is a naturally occurring human fibrin-derived peptide (Bβ15–42) generated by plasmin-mediated degradation of cross-linked fibrin [37]. In rats subjected to transient left anterior descending coronary artery occlusion with subsequent 2 h reperfusion, FX06 treatment reduced infarct size by approximately 50%, an effect comparable to that of ischemic preconditioning [38]. FX06 prevents the dissociation of intercellular VE-cadherin complexes, thereby inhibiting the last and irreversible step in leukocyte diapedesis, the actual transmigration through endothelial junctions and into the injured tissue. The compound shifts the balance of RhoA/Rac1 signaling toward endothelial junction stabilization by suppressing RhoA activity while enhancing Rac1 activity, thereby limiting stress fiber formation through inhibition of myosin light chain phosphorylation [39]. The recent F.I.R.E. (Efficacy of FX06 in the prevention of myocardial reperfusion injury) trial investigated whether early infusion (time-to-therapy < 3 h) of FX06, as an adjunct to primary PCI, would reduce the necrotic core zone of the affected myocardium by mitigating the reperfusion injury [38].

CU06-1004 is a pseudo-sugar derivative of cholesterol currently being explored across multiple disease settings associated with microvascular leakage. Its primary mechanism of action involves dual anti-inflammatory and barrier-protective effects, mediated through suppression of NF-κB signaling and enhancement of endothelial barrier integrity via Rac activation and cortactin translocation [40]. CU06-1004 exerts therapeutic effects by inhibiting vascular leakage and inflammation in various animal models, such as diabetic retinopathy, stroke, cancer, and inflammatory bowel disease [41]. Intravenous injection of CU06-1004 at the time of reperfusion ameliorated the harsh microenvironment of infarcted hearts with increased cardiomyocyte survival and capillary density as well as reduced fibrosis, resulting in improved cardiac function and adverse remodeling. Within infarcted cardiac tissue, CU06-1004 safeguarded CMEC junctional structures and sustained pericyte investment along CMECs, thereby supporting CMEC stability, function, and proliferation via bidirectional cellular interactions [42]. Despite promising preclinical results, clinical translation remains challenging because microvascular obstruction varies substantially among patients and the therapeutic window is extremely narrow, as microvascular disruption and edema develop rapidly after reperfusion, requiring agents such as FX06 and CU04-1004 to be administered very early alongside primary PCI (e.g., time-to-therapy < 3 h) to prevent irreversible leukocyte diapedesis and downstream tissue injury.

### 3.2. Second Defense Line—Spatiotemporal Modulation of Inflammatory Immune Responses

Neutrophil targeting has gained attention as a promising therapeutic strategy for I/R injury, reflecting the dynamic and context-dependent roles of neutrophils following MI [43]. Although neutrophil activation is essential for initiating the inflammatory cascade after MI, subsequent neutrophil death—particularly via the immunologically silent pathway of apoptosis—plays a central role in resolving post-MI inflammation [44]. Efferocytosis of apoptotic neutrophils by macrophages drives a phenotypic transition toward a pro-reparatory state, characterized by the release of pro-resolving lipid mediators and transforming growth factor-β (TGF-β). Taken together, the harmful impact of neutrophils during the early phases of MI outweighs their reparative contributions, underscoring the importance of suppressing excessive neutrophil-driven inflammation while fostering neutrophil-associated healing responses [45].

Inhibitors of NETosis, a form of neutrophil death involving the extrusion of NETs, have been shown to reduce cardiac injury in preclinical MI models. Neutrophil stunning by the β1-adrenergic-receptor antagonist metoprolol has been shown to reduce infarct size in acute MI patients [46]. Neutrophil-based cardioprotective strategies that minimize cardiac injury are potentially advantageous over regenerative strategies such as stem cell-based therapies, microRNA targeting therapies, or fibroblast to CM reprogramming strategies that aim at repairing an infarcted heart that has undergone largely irreversible damage [47]. However, despite the immense importance of neutrophils in the early stages of MI pathogenesis, no therapeutic strategy has heretofore been able to modulate both neutrophil-mediated inflammation and healing in a balanced and holistic manner. Several preclinical and clinical studies that downregulate neutrophil activation have failed to demonstrate therapeutic efficacy [48]. For example, antibody-mediated depletion of neutrophils worsens cardiac fibrosis and heart function in MI mice, because the lack of neutrophils within the infarcted heart reduces the efferocytosis capacity of cardiac macrophages and shifts the macrophage balance towards the pro-inflammatory phenotype [49].

Recently, roscovitine- and catalase-loaded PLGA nanoparticles (RC-NPs) were developed for optimal neutrophil tropism, enabling rapid roscovitine release in activated neutrophils within the infarcted heart, thereby allowing spatiotemporal control over neutrophil fate [50]. Roscovitine is a cyclin-dependent kinase inhibitor that effectively induces neutrophil apoptosis and has been shown to be safe and tolerable in clinical trials [51]. Intravenous injection of RC-NPs after MI result in RC-NP uptake by circulating neutrophils that subsequently infiltrate the infarcted heart. Activated neutrophils in the infarcted heart generate hydrogen peroxide (H2O2), which would be converted to oxygen by the catalase in RC-NPs. The buildup of oxygen gas causes internalized RC-NPs to detonate and rapidly release roscovitine, inducing timely apoptosis of activated neutrophils. RC-NPs would therefore mitigate cardiac damage caused by overexuberant neutrophil signaling while promoting macrophage efferocytosis of apoptotic neutrophils, subsequent macrophage polarization, and healing. Compared with small molecule- or antibody-based neutrophil modulating strategies which indiscriminately downregulate neutrophil activation at the cost of macrophage efferocytosis and healing, RC-NPs offer a more balanced mode of neutrophil modulation. Furthermore, RC-NPs would rapidly release roscovitine in activated neutrophils but release roscovitine more slowly in non-ROS conditions, reducing the risk of adverse side effects such as neutropenia and off-target toxicity. These findings provide the therapeutic potential of targeting neutrophils to tune both inflammatory and reparatory processes following MI. Despite these promising findings, a major translational challenge lies in suppressing excessive early inflammation while preserving later reparative immune responses, as indiscriminate systemic immunosuppression—such as non-specific neutrophil depletion—can disrupt macrophage efferocytosis, exacerbate fibrosis, and ultimately worsen cardiac function. Accordingly, achieving precise spatiotemporal drug delivery that restricts therapeutic activity to activated neutrophils within the infarcted myocardium, thereby minimizing systemic neutropenia and off-target toxicity, remains a key hurdle for clinical translation.

### 3.3. Third Defense Line—Antioxidants to Prevent Non-Apoptotic Programmed Cell Death

Under homeostatic conditions, ROS function as second messengers regulating cell survival, growth, and intracellular signaling [52]. However, during myocardial I/R injury, excessive ROS accumulation drives DNA damage, protein denaturation, and lipid perox-idation [13,15,53,54]. Antioxidants are molecules that attenuate or interrupt ROS-driven chain reactions even at very low concentrations. Among them, superoxide dismutase, cat-alase, and glutathione peroxidase constitute the primary line of defense against excessive ROS accumulation [55]. Several antioxidant-based compounds such as resveratrol and high-dose intravenous vitamin C, have been explored as adjunctive cardioprotective strategies, with preclinical benefits largely attributed to modulation of oxidative stress and inflammatory signaling [56,57]. However, their clinical translation has yielded limited patient benefit, largely because these approaches have primarily focused on caspase-dependent apoptosis as the therapeutic target. 

Unlike broad oxidative damages, ferroptosis is characterized by highly specific, iron-catalyzed massive lipid peroxidation. During severe I/R injury, iron overload further amplifies this process through Fenton chemistry and enzymatic lipid oxidation, while impaired glutathione-dependent repair systems promote lethal lipid ROS accumulation, establishing ferroptosis as a key non-apoptotic mechanism of injury [14,15]. In our pre-vi-ous study, we demonstrated the therapeutic role of Histochrome^®^ as a ferroptosis inhi-bi-tor in myocardial I/R injury [58]. Histochrome^®^ has potent antioxidant effects and is known as an iron chelator [59]. Histochrome^®^ has been shown to improve GSH levels and increase GPX4 expression, thereby suppressing iron overload and ferroptosis. This drug has demonstrated clinical safety and cardioprotective efficacy in human patients when administered prior to thrombolysis in acute MI and prior to coronary artery bypass graft-ing in ischemic heart disease. It is already used in cardiology practice with a 1% Histo-chrome^®^ solution via boluses or droppers and has exhibited no serious complications re-quiring special correction: no changes were observed in the functional parameters of liver and kidneys, no variations were found in blood indexes (hemoglobin and hematocrit), and no allergic reactions [60]. These data support the safety profile of Histochrome^®^ for myocardial protection during reperfusion-related interventions. Despite the compelling mechanistic rationale and promising efficacy of anti-ferroptotic interventions, it is crucial to acknowledge that the definitive clinical relevance of ferroptosis in human myocardial infarction remains largely unproven until now. Until robust clinical diagnostics can con-firm the specific contribution of ferroptosis to infarct expansion in human STEMI patients, targeting this pathway remains an experimental, albeit highly promising, frontier rather than an established clinical reality.

Single-atom nanozymes have emerged as next-generation antioxidant platforms that overcome the limited stability and rapid consumption of conventional antioxidants [61]. Importantly, Pt-doped ceria nanozymes (Pt@CeNZ) demonstrate preferential uptake and prolonged intracellular retention in cardiomyocytes, leading to sustained intracellular antioxidant effects [62]. Under I/R-related oxidative stress, Pt@CeNZ effectively suppresses acute ROS accumulation and preserves cardiomyocyte viability. Beyond conventional ROS neutralization, Pt@CeNZ also inhibits ferroptosis by attenuating iron-dependent lipid peroxidation. This dual mechanism allows Pt@CeNZ to regulate non-apoptotic programmed cell death pathways that are highly relevant to myocardial I/R injury. In preclinical models, Pt@CeNZ treatment results in marked attenuation of oxidative damage within ischemic myocardium. These effects translate into reduced infarct size and improved post-reperfusion cardiac function. Overall, Pt@CeNZ represents a promising cell-free antioxidant strategy targeting both oxidative stress and ferroptosis in myocardial I/R injury. Collectively, these therapeutic strategies illustrate emerging approaches aimed at interrupting interconnected pathological networks triggered by I/R injury, including endothelial barrier disruption, dysregulated immune activation, oxidative stress, and ferroptosis. These therapeutic intervention points and their mechanistic targets are schematically summarized in Figure 2.

## 4. Conclusions

Myocardial I/R injury should be viewed as a dynamic process driven by interdependent metabolic, inflammatory, microvascular, and regulated cell death pathways. Rather than representing a single pathological event, reperfusion can be viewed as a dynamic transition state in which restoration of blood flow converts ischemic stress into a self-amplifying injury network that shapes infarct evolution and adverse remodeling [63]. Although timely reperfusion remains the cornerstone of STEMI management, the abrupt biological transitions occurring during reperfusion continue to propagate myocardial damage despite successful restoration of coronary perfusion. Accumulating evidence indicates that these processes do not operate in isolation, which likely explains the limited clinical success of previous single-target cardioprotective strategies. Emerging therapeutic approaches therefore increasingly aim to simultaneously modulate multiple components of reperfusion injury, including endothelial barrier integrity, innate immune amplification, oxidative stress, and regulated cell death pathways. Importantly, the effectiveness of such interventions will depend not only on mechanistic targeting but also on precise temporal alignment with the evolving phases of post-ischemic inflammation and repair, highlighting the need for therapies that are matched to the shifting balance between injury propagation and tissue regeneration during reperfusion.

Future translational progress will require integrative strategies that combine mechanistic insight, spatiotemporal therapeutic control, and clinically relevant biomarkers to bridge the gap between experimental cardioprotection and successful patient outcomes. Ultimately, meaningful clinical progress will likely come not from targeting isolated pathways, but from strategically integrating therapies that address the dynamic and interconnected nature of reperfusion injury.

## Figures and Tables

**Table 1:** Comparative overview of current clinical and emerging experimental therapeutic strategies targeting myocardial I/R injury.

Category	Therapeutic Strategy	Representative Examples	Mechanistic Focus	Clinical/Translational	Key Translational Limitations
**Clinical practice**	Antiplatelet therapy	Aspirin, P2Y12 inhibitors	Suppression of platelet activation and thrombo-inflammatory signaling	Standard of care in ACS-STEMI	Residual microvascular injury despite optimal platelet inhibition
	Antioxidant-based therapy	Histochrome^®^, Resveratrol, Vitamin C	Iron chelation, ROS reduction, ferroptosis modulation	Clinical use (Histochrome^®^), Clinical studies (Resverartol, Vitamin C)	Limited large-scale randomized evidence
**Experimental (preclinical)**	Endothelial barrier protection	CU06-1004	NF-κB inhibition, endothelial stabilization, reduced vascular leakage	Preclinical	Narrow therapeutic timing window; patient heterogeneity
	Spatiotemporal immune modulation	Roscovitine/catalase-loaded nanoparticles (RC-NPs)	Selective neutrophil apoptosis and immune reprogramming	Preclinical	Balancing early inflammation suppression vs. later repair
	Nanozyme-based antioxidant therapy	Pt@CeNZ	Sustained ROS scavenging and ferroptosis inhibition	Preclinical	Delivery efficiency and long-term safety validation
**Experimental (conceptual)**	Multi-target network modulation	Combination or systems-level approaches	Simultaneous control of oxidative stress, inflammation, and microvascular dysfunction	Emerging concept	Complexity of clinical trial design and biomarker-guided patient selection

## Data Availability

No new data were created or analyzed in this study. Data sharing is not applicable to this article.

## Abbreviations

The following abbreviations are used in this manuscript:

I/R	Ischemia–Reperfusion
IHD	Ischemic Heart Disease
MI	Myocardial Infarction
PCI	Percutaneous Coronary Intervention
STEMI	ST-Segment Elevation Myocardial Infarction
mPTP	Mitochondrial Permeability Transition Pore
CMEC	Cardiac Microvascular Endothelial Cells
RC-NPs	Roscovitine- and Catalase-Loaded PLGA Nanoparticles
PUFAs	Polyunsaturated Fatty Acids
Pt@CeNZ	Pt-Doped Ceria Nanozymes
